# A matched-pair analysis comparing whole-brain radiotherapy with and without a stereotactic boost for intracerebral control and overall survival in patients with one to three cerebral metastases

**DOI:** 10.1186/s13014-017-0804-1

**Published:** 2017-04-24

**Authors:** Dirk Rades, Stefan Janssen, Amira Bajrovic, Mai Trong Khoa, Theo Veninga, Steven E. Schild

**Affiliations:** 1 Department of Radiation Oncology, University of Lübeck, Ratzeburger Allee 160, 23538 Lübeck, Germany; 2 Medical Practice for Radiotherapy and Radiation Oncology, Hannover, Germany; 3 Department of Radiation Oncology, University Medical Center Eppendorf, Hamburg, Germany; 4 Nuclear Medicine and Oncology Center, Bach Mai Hospital, Hanoi, Vietnam; 5 Department of Radiotherapy, Dr. Bernard Verbeeten Institute, Tilburg, Netherlands; 6 Department of Radiation Oncology, Mayo Clinic, Scottsdale, AZ USA

**Keywords:** Cerebral metastases, Whole-brain radiotherapy, Stereotactic boost, Intracerebral control, Overall survival

## Abstract

**Background:**

Twelve years ago, a randomized trial demonstrated that a radiosurgery boost added to whole-brain radiotherapy (WBRT) improved intracerebral control (IC) in patients with one to three cerebral metastases. Overall survival (OS) was improved only in the subgroup of patients with a single metastasis but not in the entire cohort. The present study compared both regimens in a different scenario outside a randomized trial.

**Methods:**

A total of 252 patients with one to three cerebral metastases were included. Eighty-four patients receiving WBRT plus a planned stereotactic boost and 168 patients receiving WBRT alone were individually matched 1:2 for nine factors including fractionation of WBRT, age, gender, performance score, primary tumor, number of cerebral metastases, extracerebral metastases, recursive partitioning analysis class, and time between cancer diagnosis and WBRT. Each group of three patients was required to match for all nine factors. Both groups were compared for IC and OS.

**Results:**

IC rates at 6, 12, 18 and 24 months were 88, 71, 45 and 22% after WBRT plus stereotactic boost vs. 75, 48, 38 and 22% after WBRT alone (*p* = 0.005). OS rates at 6, 12, 18 and 24 months were 76, 53, 32 and 25% after WBRT plus stereotactic boost and 67, 45, 29 and 20% after WBRT alone (*p* = 0.10). In patients with a single lesion, OS rates were also not significantly different (*p* = 0.12).

**Conclusions:**

Similar to the previous randomized trial from 2004, this matched-pair study showed that a stereotactic boost in addition to WBRT significantly improved IC but not OS.

## Introduction

About 40% of all cancer patients who developed cerebral metastases present with up to three lesions [1, 2]. These patients have a more favorable survival than those patients with more metastases. It has been suggested that patients with up to three lesions may benefit from local therapies such as neurosurgical resection, stereotactic radiosurgery (SRS) and fractionated stereotactic radiotherapy (FSRT) [2]. The optimal treatment approach is yet to be defined. Several randomized trials demonstrated that WBRT plus a stereotactic boost resulted in better intracerebral control (IC) than SRS alone without improving overall survival (OS) [3–6]. Since both intracerebral recurrence and WBRT can decrease neurocognitive function, it is not absolutely clear, whether SRS alone is appropriate for one to three cerebral metastases or whether WBRT plus SRS is best [7–9]. Only two randomized trials compared SRS plus WBRT to WBRT alone [10, 11]. Both were published more than 12 years ago. One trial was stopped early after inclusion of 27 patients [10], and the other (RTOG 9508) was completed after inclusion of 333 patients [11]. Thus, there is only one trial with adequate statistical power that compared SRS plus WBRT and WBRT [11]. According to its results, an SRS boost significantly improved intracerebral control (IC). OS was not improved for the entire cohort but in the subgroup of patients with a single metastasis. The present study compared WBRT plus a planned stereotactic boost to WBRT alone in a different scenario outside a randomized trial with respect to OS and IC, which was defined as freedom from a recurrence anywhere in the brain. The study was performed as a matched-pair-analysis, where patients of the two treatment groups were required to individually match for nine factors. Since another randomized trial would be difficult to perform, a matched-pair study is the best alternative design to reduce the risk of selection biases.

## Patients and methods

A total of 252 patients with a Karnofsky performance score (KPS) of ≥70 were irradiated for one to three newly diagnosed cerebral metastases (size ≤4 cm) from 1998 to 2014 and included in this matched-pair study. The diagnosis of cerebral metastases was confirmed by magnetic resonance imaging (MRI). Eighty-four patients received WBRT followed by a planned stereotactic boost to the metastatic lesions and 168 patients received WBRT alone. These patients were part of an anonymized database of 2160 patients irradiated for brain metastases. Treatment did depend on institutional preferences and physicians’ choices at certain periods of time. Patients of both treatment groups were individually matched 1:2 with respect to nine characteristics/potential prognostic factors, which was done to decrease biases due to imbalances of these potential prognostic factors. These characteristics included fractionation of WBRT (5x4 Gy in 1 week vs. longer-course WBRT, i.e. 10x3 Gy in 2 weeks or 20x2 Gy in 4 weeks), age at the time of WBRT (≤58 years vs. ≥59 years, median age = 58 years), gender, KPS (70 vs. ≥80), type of primary tumor (breast cancer vs. lung cancer vs. other cancers), number of cerebral metastases (single vs. multiple, i.e. 2–3), extracerebral metastases (no vs. yes), recursive partitioning analysis (RPA) class (1 vs. 2), and time interval between cancer diagnosis and start of WBRT (≤15 months vs. ≥16 months, median 16 months). Patients treated in the Netherlands received 5x4 Gy, and those patients treated in Germany longer-course WBRT. Thus, 5x4 Gy was not given preferentially to patients with a poor prognosis. Median size of the treated lesions was 11 mm (range: 5–32 mm) in the WBRT plus SRS group and 14.5 mm (range: 4–40 mm) in the WBRT alone group, respectively. Distributions of these characteristics are summarized in Table 1.

**Table 1:** Distribution of the patient characteristics/potential prognostic factors in both treatment groups

	WBRT + stereotactic boost (*n* = 84) N patients (%)	WBRT alone (*n* = 168) N patients (%)
Fractionation of WBRT		
5 x 4 Gy (*n* = 33)	11 (13)	22 (13)
Longer-course WBRT (*n* = 219)	73 (87)	146 (87)
Age at WBRT		
≤58 years (*n* = 132)	44 (52)	88 (52)
≥ 59 years (*n* = 120)	40 (48)	80 (48)
Gender		
Female (*n* = 150)	50 (60)	100 (60)
Male (*n* = 102)	34 (40)	68 (40)
Karnofsky performance score		
70 (*n* = 78)	26 (31)	52 (31)
≥80 (*n* = 174)	58 (69)	116 (69)
Type of primary tumor		
Breast cancer (*n* = 69)	23 (27)	46 (27)
Lung cancer (*n* = 156)	52 (62)	104 (62)
Other cancers (*n* = 27)	9 (11)	18 (11)
Number of cerebral metastases		
1 (*n* = 120)	40 (48)	80 (48)
2-3 (*n* = 132)	44 (52)	88 (52)
Extracerebral metastases		
No (*n* = 114)	38 (45)	76 (45)
Yes (*n* = 138)	46 (55)	92 (55)
RPA class		
Class 1 (*n* = 90)	30 (36)	60 (36)
Class 2 (*n* = 162)	54 (64)	108 (64)
Interval from cancer diagnosis to WBRT		
≤ 15 months (*n* = 120)	40 (48)	80 (48)
≥ 16 months (*n* = 132)	44 (52)	88 (52)

WBRT was performed with 6–10 MV photon beams from a linear accelerator. The stereotactic boost was administered with a linear accelerator in 76 patients (dose prescribed to the margin of the metastatic lesions, representing the 80–90% isodose line) and with a GammaKnife in eight patients (dose prescribed to the margin of the metastatic lesions, representing the 50–60% isodose line). The Gamma Knife treatment was performed as single-fraction radiosurgery in all eight patients. In those 76 patients receiving their stereotactic boost from a linear accelerator, 59 received single-fraction radiosurgery, and 17 patients fractionated stereotactic radiotherapy (FSRT) with two to five fractions of 4–8 Gy. FSRT was generally administered in patients with at least one metastatic lesion located in or close to the brainstem or other critical structures. The median dose of radiosurgery was 20 Gy (range: 15–25 Gy), and the median total dose of FSRT 18 Gy (range: 12–40 Gy).

The two treatment groups were compared for IC and OS. IC was defined as freedom from development of new cerebral metastases and progression of the treated cerebral lesions. New and progressive cerebral metastases were identified by magnetic resonance imaging performed at regular intervals (3 to 4 months) and for progressive clinical symptoms. Both IC and OS were referenced from the start of radiotherapy. Univariate analyses for both endpoints were performed with the Kaplan-Meier method supplemented by the Wilcoxon test to determine the differences between the corresponding curves [12]. *P*-values of <0.05 were considered significant. Additional multivariate analyses were not required, since the patients of both treatment groups were individually matched 1:2 taking into account nine factors. Each group of three patients matched for all factors. Thus, the risk of a selection bias was lower with this method of than with the method of propensity score matching.

## Results

**Fig. 1:**
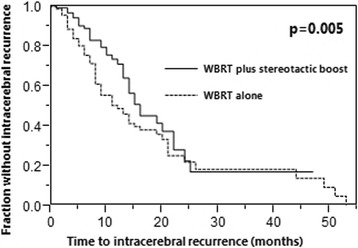
Comparison of the two treatment groups WBRT plus stereotactic boost and WBRT alone with respect to intracerebral control

Patients were followed until death or for a median of 11 months (range: 4–54 months). In the entire cohort, the IC rates at 6, 12, 18 and 24 months were 80, 56, 40 and 22%, respectively. IC rates after WBRT plus a stereotactic boost were 88, 71, 45 and 22%, respectively, versus 75, 48, 38 and 22%, respectively, after WBRT alone (*p* = 0.005, Fig. 1).

**Fig. 2:**
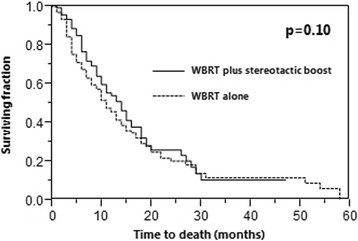
Comparison of the two treatment groups WBRT plus stereotactic boost and WBRT alone with respect to overall survival

Median survival times in the entire cohort, the WBRT plus stereotactic boost group and the WBRT alone group were 11, 14 and 11 months, respectively. In the entire cohort, OS rates at 6, 12, 18 and 24 months were 70, 48, 30 and 22%, respectively. OS rates after WBRT plus a stereotactic boost were 76, 53, 32 and 25%, respectively, vs. 67, 45, 29 and 20%, respectively, after WBRT alone (*p* = 0.10, Fig. 2).

In accordance with a previous randomized trial [11], a subgroup analysis for OS was performed in the 120 patients with a single cerebral lesion. In this subgroup, the OS rates at 6, 12, 18 and 24 months were 72, 54, 30 and 23%, respectively. OS rates after WBRT plus a stereotactic boost were 83, 64, 34 and 30%, respectively, vs. 67, 49, 29 and 18%, respectively, after WBRT alone (*p* = 0.12).

## Discussion

Most cancer patients with one to three cerebral metastases are treated with radiotherapy alone. Several radiotherapy approaches exist including WBRT alone, SRS or FSRT alone, and WBRT plus a stereotactic boost (SRS or FSRT). Several randomized trials compared SRS alone to SRS plus WBRT [3–6]. Aoyama et al. presented a trial of 132 patients with one to four cerebral metastases, 67 treated with SRS and 65 with SRS plus WBRT [3]. WBRT significantly improved IC at 12 months (53% vs. 24%, *p* < 0.001) but not OS (39% vs. 28%, *p* = 0.42). In 2009, Chang et al. reported a randomized trial of 58 patients with one to three cerebral metastases that was stopped after an interim analysis, since the decline in neurocognitive function at 4 months was significantly worse in the SRS plus WBRT group than in the SRS alone group (mean posterior probability of decline 52% vs. 24%) [4]. IC rates at 12 months were 73% after SRS plus WBRT and 27% after SRS alone (*p* < 0.001). Unfortunately, neurocognitive function was not assessed at 12 months. OS rates were not significantly different. In the third randomized trial comparing SRS plus WBRT to SRS alone, both SRS and neurosurgical resection were allowed and compared to the same procedures plus WBRT. In the subgroup of 199 patients treated with SRS, 100 patients received SRS alone and 99 patients SRS plus WBRT. WBRT significantly improved local control of the treated lesions (*p* = 0.040) and freedom from new cerebral metastases (*p* = 0.008) at 2 years. Similar to the previous two trials, OS was not significantly improved with WBRT. In the most recent trial of 213 patients with one to three cerebral metastases, cognitive progression at 3 months was significantly more severe in the SRS plus WBRT group. Time to intracranial failure was significantly shorter after SRS alone than after SRS plus WBRT (*p* < 0.001). Median OS times were 10.4 months vs. 7.4 months (*p* = 0.92). When summarizing these trials, it appears that WBRT in addition to SRS significantly improves IC but not OS [3–6]. Unfortunately, WBRT also leads to more pronounced neurocognitive dysfunction. However, an intracerebral recurrence can be associated with impairment of neurocognitive function. Thus, the omission of WBRT may not be best in all situations. A recent meta-analysis of three trials [3–5] suggested omitting WBRT in patients aged ≤50 years [13].

The risk of developing neurocognitive decline can be reduced by using doses per fraction considerably lower than 3 Gy and avoiding concomitant chemotherapy [14, 15]. Furthermore, hippocampus sparing WBRT offers an additional approach to reduce the risk of neurocognitive decline. In the study of Gondi et al., who used the Hopkins Verbal Learning Test - Revised for Delayed Recall (HVLT-R DR), the rates of decline at 4 months vs. baseline were 7% with hippocampal sparing and 30% in a historic control group (*p* < 0.001) [16]. In a randomized trial of 508 eligible patients, memantine in addition to WBRT (20 mg/d, starting 3 days prior to WBRT for 24 weeks) showed a strong trend towards less decline in delayed recall at 24 weeks when compared to WBRT without memantine (*p* = 0.059). Significantly better results were found for several endpoints including executive function at 16 weeks (*p* = 0.008), processing speed at 24 weeks (*p* = 0.014) and delayed recognition at 24 weeks (*p* = 0.015).

When using these new options to lower the risk of WBRT-induced neurocognitive decline, WBRT should still be considered an option for patients with one to three cerebral metastases. In this light, one important question is whether results of WBRT can be improved with a stereotactic boost? Two randomized trials were reported so far that compared WBRT with and without a stereotactic boost [10, 11]. One small trial of patients with two to four lesions was stopped after inclusion of 27 patients [10]. One-year local failure rates were 8% after WBRT plus SRS and 100% after WBRT alone (*p* = 0.002). Median OS times were not significantly different (11 vs. 7.5 months, *p* = 0.22). The other randomized trial included 331 eligible patients with one to three cerebral metastases and a KPS of ≥70 (RPA class 1 or 2) [11]. In this trial, SRS in addition to WBRT significantly improved local control of the treated lesions at 1 year (82% vs 71%, *p* = 0.01). IC, which was defined as increase in size of any lesion, new cerebral lesions or neurological deterioration despite stable disease on MRI, was also significantly improved (*p* = 0.013). However, IC rates were not explicitly stated. An OS benefit was limited to patients with a single cerebral metastasis. Median survival times were 6.5 and 4.9 months, respectively (*p* = 0.039), whereas in the entire cohort OS was not significantly different (6.5 vs 5.7 months, *p* = 0.14).

Similar to the previous trial (*p* = 0.013), the addition of a stereotactic boost significantly improved IC in the present study (*p* = 0.005) [11]. OS was not significantly affected in the entire series, which agreed well with the results of the two previous randomized studies [10, 11]. In the RTOG 9508 trial, an OS benefit was found in patients with a single cerebral metastasis. This result was not confirmed in the present study, although a certain trend was observed (*p* = 0.12). One may speculate whether the number of 120 patients with a single lesion in the present study was too small to provide adequate statistical power to detect a significant difference. The number of patients with a single lesion in the RTOG 9508 trial was greater (*n* = 186). Furthermore, although this matched-pair study followed very strict matching criteria (individual matching for nine factors) the source of data still is retrospective in nature. In case of retrospective data, the risk of a hidden selection bias always remains. One potential selection bias may have been introduced due to the difference regarding the size of the treated lesions that were slightly smaller in the WBRT plus SRS group.

When reflecting the results of the two randomized trials [10, 11] and the present study, it appears that a stereotactic boost in addition to WBRT significantly improves local and intracerebral control. Since an intracerebral recurrence may lead to severe symptoms and even death, a stereotactic boost can be recommended for patients with one to three cerebral metastases selected for WBRT. This recommendation is supported by other results of the RTOG 9508 trial [11]. The stereotactic boost led to improved functional autonomy in all patients and improved OS in patients with a single lesion. The recommendation applies to the majority of patients with few cerebral metastases. In a study that developed a score to predict the probability of developing new metastases after SRS alone, only 22% (47/214) of patients were considered good candidates SRS alone [17]. When adding a stereotactic boost to WBRT two additional aspects need to be considered. A stereotactic boost will likely be associated with an increased risk of radionecrosis, particularly when combined with modern targeted therapies such as ipilimumab that are increasingly used in cancer patients [18, 19]. Another aspect, which needs to be considered, is cost-effectiveness [18]. In a cost-effectiveness analysis published in 2015 that compared neuro-cognitive sparing radiotherapy programs to WBRT alone in patients with one to three brain metastases, hippocampal sparing WBRT plus a stereotactic boost proved to be cost-effective when compared to WBRT alone in patient groups with a median survival pf 12 months or longer following radiotherapy [20]. Moreover, WBRT plus a boost to the metatatic sites can be safely perfomed with volumetric modulated arc therapy (VMAT) in one course as WBRT plus a simultaneous integrated boost (SIB) [21].

## Conclusion

A stereotactic boost in addition to WBRT resulted in significantly better IC than WBRT alone without significantly improving OS. Since an intracerebral recurrence can cause major symptoms and even death, IC must be considered an important goal. Therefore, the addition of a stereotactic boost to WBRT should be considered for patients with very few cerebral metastases, who were identified as candidates for WBRT and not for SRS alone.
